# Comprehensive circRNA-microRNA-mRNA network analysis revealed the novel regulatory mechanism of *Trichosporon asahii* infection

**DOI:** 10.1186/s40779-021-00311-w

**Published:** 2021-03-09

**Authors:** Ming-Wang Zhang, Zhi-Hong Zhu, Zhi-Kuan Xia, Xin Yang, Wan-Ting Luo, Jun-Hong Ao, Rong-Ya Yang

**Affiliations:** 1 Department of Dermatology, Southwest Hospital, Army Medical University, Chongqing, 400038 China; 2 Department of Dermatology, The Seventh Medical Center of PLA General Hospital, 5 Nanmencang, Dongcheng District, Beijing, 100700 China; 3 Department of Ophthalmology, Hainan Hospital of Chinese PLA General Hospital, Sanya, 572000 Hainan China; 4 The Second School of Clinical Medicine, Southern Medical University, Guangzhou, 510280 Guangdong China

**Keywords:** *Trichosporon asahii*, Circular RNA, Competing endogenous RNA, RNA sequencing

## Abstract

**Background:**

Invasive *Trichosporon asahii* (*T. asahii*) infection frequently occurs with a high mortality in immunodeficient hosts, but the pathogenesis of *T. asahii* infection remains elusive. Circular RNAs (circRNAs) are a type of endogenous noncoding RNA that participate in various disease processes. However, the mechanism of circRNAs in *T. asahii* infection remains completely unknown.

**Methods:**

RNA sequencing (RNA-seq) was performed to analyze the expression profiles of circRNAs, microRNAs (miRNAs), and mRNAs in THP-1 cells infected with *T. asahii* or uninfected samples. Some of the RNA-seq results were verified by RT-qPCR. Gene Ontology (GO) enrichment and Kyoto Encyclopedia of Genes and Genomes (KEGG) pathway analyses were used to analyze the differentially expressed mRNAs. A circRNA-miRNA-mRNA network was constructed and verified by dual-luciferase reporter assay and overexpression experiments.

**Results:**

A total of 46 circRNAs, 412 mRNAs and 47 miRNAs were differentially expressed at 12 h after *T. asahii* infection. GO and KEGG analyses showed that the differentially expressed mRNAs were primarily linked to the leukocyte migration involved in the inflammatory response, the Toll-like receptor signaling pathway, and the TNF signaling pathway. A competing endogenous RNA (ceRNA) network was constructed with 5 differentially expressed circRNAs, 5 differentially expressed miRNAs and 42 differentially expressed mRNAs. Among them, hsa_circ_0065336 was found to indirectly regulate PTPN11 expression by sponging miR-505-3p.

**Conclusions:**

These data revealed a comprehensive circRNA-associated ceRNA network during *T. asahii* infection, thus providing new insights into the pathogenesis of the *T. asahii*-host interactions.

**Supplementary Information:**

The online version contains supplementary material available at 10.1186/s40779-021-00311-w.

## Background

*Trichosporon asahii (T. asahii)*, the most common pathogenic species in the genus Trichosporon, which is widely distributed in tropical and temperate regions, usually invades the human body through internal catheters, the intestinal mucosa translocation of microorganisms or respiratory inhalation [1]. *T. asahii* infection is usually associated with superficial mycosis in immunocompetent hosts but can cause invasive infections in immunosuppressed patients. With the abuse of immunosuppressants and broad-spectrum antibiotics and the extensive development of traumatic surgery, the incidence of invasive trichosporonosis has increased over the years [2]. Although various antifungal drugs such as amphotericin B, flucytosine, caspofungin and fluconazole have been used to treat invasive trichosporonosis, the mortality associated with invasive trichosporonosis is still high and requires further analysis [3].

Circular RNAs (circRNAs), a type of endogenous noncoding RNA that are widely distributed in eukaryotic cells and have relatively conserved regulatory functions and tissue-specific and cell-specific expression patterns [4]. Although circRNAs were discovered more than 40 years ago, they were initially mainly regarded as “junk” generated by mis-splicing of transcripts [5, 6]. In recent years, with the wide application of RNA sequencing (RNA-seq) technology and the rapid development of circRNA-specific computational tools, various circRNAs have been reported to be involved in the pathogenesis of cancer, neurological disorders, cardiovascular diseases, diabetes mellitus and autoimmune diseases [7–11]. However, circRNAs associated with fungal infections have not been reported to date.

MicroRNAs (miRNAs) are small noncoding RNAs that can negatively control their target gene expression post-transcriptionally [12, 13]. Recent studies have characterized miRNA expression profiles and identified several critical miRNAs that participate in immune and inflammatory responses following fungal exposure [14]. However, the upstream regulation mechanisms of miRNAs are poorly understood. It has been reported that circRNAs can regulate gene expression by binding miRNA response elements (MREs) as competing endogenous RNAs (ceRNAs) [15]. However, whether circRNAs serve as ceRNAs and are involved in the interactions between *T. asahii* and its hosts remain unknown.

In this study, we systematically investigated alterations in the circRNA, miRNA, and mRNA expression profiles of macrophages during *T. asahii* infection by using RNA-seq. A large number of dysregulated circRNAs, miRNAs and mRNAs were identified after *T. asahii* infection. The ceRNA network revealed the putative roles of circRNAs in regulating the cellular response to *T. asahii* infection. Furthermore, we found that hsa_circ_0065336 could indirectly regulate PTPN11 expression by sponging miR-505-3p. In summary, this is the first report to provide the expression profiles and results of functional analyses of circRNAs after fungal exposure in mammalian cells.

## Methods

### Cell culture and *T. asahii* infection

The human monocyte line THP-1 was cultured and maintained in RPMI 1640 medium (Gibco, USA) supplemented with 10% fetal bovine serum (FBS) (Gibco, USA), 1% penicillin/streptomycin (Gibco, USA) and 0.05 mmol/L β-mercaptoethanol (Amresco, USA) at 37 °C and a 5% CO_2_ atmosphere. Two hundred ninety-three T cells were cultured with 10% FBS at 37 °C and 5% CO_2_. Phorbol 12-myristate 13-acetate (PMA) (Sigma, USA) at 100 ng/ml was added for 48 h to induce the differentiation of THP-1 cells into macrophages, this process was used throughout the study. Preparation of the heat-inactivated *T. asahii* standard strain *CBS2479* was performed similar to a previous description by Wu et al. [16]. In brief, *T. asahii* was collected from yeast extract peptone dextrose (YPD) liquid medium, washed twice with phosphate-buffered saline (PBS), resuspended in the same buffer, and then inactivated by heating at 65 °C for 3 h in a water bath. The efficiency of heat inactivation was confirmed by incubating the suspension in YPD agar medium for 24 h at 35 °C. THP-1 cells were mock-infected or infected with *T. asahii* at a multiplicity of infection (MOI) of 5:1.

### RNA extraction, library construction and sequencing

Total RNA was extracted from both mock-infected and *T. asahii*-infected cells at 12 h with TRIzol reagent (Invitrogen, USA) according to the manufacturer’s protocol. The concentration and purity of the total RNA were analyzed by a NanoDrop™ 2000 (Thermo Fisher Scientific, USA) and the integrity was evaluated by an Agilent 2100 bioanalyzer (Agilent Technologies, CA, USA). We constructed the following two libraries from six samples for sequencing: (1) rRNA-depleted RNA library: rRNA was removed from the total RNA (5 μg) using a Ribo-off rRNA Depletion Kit (Vazyme, China). After rRNA-depleted RNA purification, cDNA libraries were constructed using the VAHTSTM Stranded mRNA-seq Library Prep Kit for Illumina® (Vazyme, China) according to the manufacturer’s instructions. The Illumina HiSeq XTen platform (Sangon Biotech, China) was used to perform sequencing analysis. (2) miRNA library: Total RNA was collected and quantified with a Qubit 2.0 RNA Detection Kit. The 3′ and 5′ adapters were ligated by using T4 RNA Ligase 2 (New England Biolabs, USA) and T4 RNA Ligase 1 (New England Biolabs, USA), respectively, followed by reverse transcription and PCR amplification of the ligation products. The 140–150 bp PCR products were enriched and quantified with a Qubit 2.0 DNA Kit. Finally, the cDNA library was sequenced on the Illumina HiSeq XTen platform (Sangon Biotech, China).

### circRNA identification and characterization

The raw sequencing data were filtered with Trimmomatic software to obtain high-quality clean data [17]. The quality control sequences were mapped to the human reference genome hg38 using BWA-MEM [18] and then subjected to CIRI2 to identify circRNAs [19]. BEDtools was used to determine the source of circRNAs based on circRNA position and gene annotation information [20].

### Quantification of abundance and differential expression analysis

mRNA abundance was detected by the StringTie software and normalized to transcripts per million (TPM) [21]. The formula used to calculate TPM is as follows: ${TPM}_i=\frac{X_i}{L_i}\ast \frac{1}{\sum_j\frac{X_j}{L_j}}\ast {10}^6,{\mathrm{X}}_{\mathrm{i}}=\frac{total\ exon\ fragment}{reads},{L}_i=\frac{exon\ length}{KB}$. differentially expressed Seq2 was used for differential expression analysis of mRNAs [22]. A *q*-value < 0.01, |log_2_fold change (FC)| > 1 and mean TPM > 5 in at least one group were set as criteria to define significantly differentially expressed mRNAs. circRNA expression was quantified based on the number of back-spliced junction read pairs, and circRNA length was calculated as reads per kilobase of transcript per million mapped reads (RPKM). The following formula was used: $\mathrm{RPKM}=\frac{Total\ Exon\ Reads}{Mapped\ Reads\ (millions)\ast Exon\ Length\ (kb)}$. |FC| > 1.5 and *P* < 0.05 were used to indicate significant differential expression. miRNA levels were analyzed, and counts were normalized to reads per million (RPM) as follows: $\mathrm{RPM}=\frac{Exon\ Mapped\ Reads\ast {10}^6}{Total\ Mapped\ Reads}$. The miRNAs were considered differentially expressed only when |log_2_ FC| > 1 and *P* < 0.05.

### RT-qPCR validation

To verify the RNA-seq results, we performed RT-qPCR analysis of some of the differentially expressed circRNAs, miRNAs and mRNAs. Total RNA was extracted from *T. asahii*-infected and uninfected cells, and reverse transcribed to cDNA using a PrimeScript™ RT Reagent Kit with gDNA Eraser (Perfect Real Time) (Takara, Japan) according to the manufacturer’s instructions. qPCR was performed on an ABI StepOnePlus™ Real-Time PCR System (Applied Biosystems, USA). Divergent primers encompassing the back-splicing junctions for circRNAs and the convergent primers for mRNAs and miRNAs were synthesized by Sangon Biotech (China), and the sequences are shown in Table S1. β-actin was used as an internal control for circRNAs and mRNAs, and U6 served as the endogenous control for miRNAs. We used the 2^-△△Ct^ method to analyze the data. Each experiment was repeated in triplicate.

### Gene ontology (GO) enrichment and Kyoto encyclopedia of genes and genomes (KEGG) pathway analyses

GO enrichment and KEGG pathway analyses were performed to determine the role of the differentially expressed mRNAs and ceRNA network-associated target mRNAs. A *P*-value < 0.05 was used as the threshold to indicate a significant difference.

### ceRNA network analysis

A circRNA-miRNA-mRNA network was constructed according to the following standards: (1) the dataset included only differentially expressed circRNAs, miRNAs and mRNAs; (2) the expression of circRNAs/mRNAs and miRNAs followed opposite trends; (3) a Pearson correlation coefficient (*r*) > 0.8 between circRNAs and mRNAs was set as a criterion; and (4) the co-expressed circRNA-mRNA pairs had the same putative miRNA binding sites based on miRanda [23]. Cytoscape software (v3.8.0) was used to construct and visualize the ceRNA network [24].

### Cell transfection

The hsa_circ_0065336 sequence was cloned into the pCD5-ciR overexpression vector (Geneseed, Guangzhou, China). miR-505-3p mimics (5′-CGUCAACACUUGCUGGUUUCCU-3′) and its negative control (NC) (5′-ACUACUGAGUGACAGUAGA-3′) were purchased from RiboBio (Guangzhou, China). THP-1 cells were transfected using Lipofectamine 2000 (Invitrogen, CA, USA) and then co-cultured with heat-inactivated *T. asahii* for 12 h at a MOI of 5:1.

### Dual-luciferase assay

The sequences of hsa_circ_0065336 and the 3′-UTR of PTPN11 with wild-type (WT) and mutant (MUT) miR-505-3p binding site were amplified and cloned into pmir-GLO (Promega, Madison, WI, USA). miR-505-3p mimics or NC was co-transfected into 293 T cells using Lipofectamine 2000 (Invitrogen, CA, USA). At 48 h after transfection, the luciferase activity was detected by the Dual-Glo Luciferase Assay System (Promega, Madison, WI, USA) following the manufacturer’s instructions.

### Statistical analyses

The measured data are presented as the means ± standard deviations (SDs). Statistical significance between different groups was examined using Student’s *t*-test. A *P*-value < 0.05 was used to indicate statistical significance.

## Results

### Identification and characterization of circRNAs

**Fig. 1:**
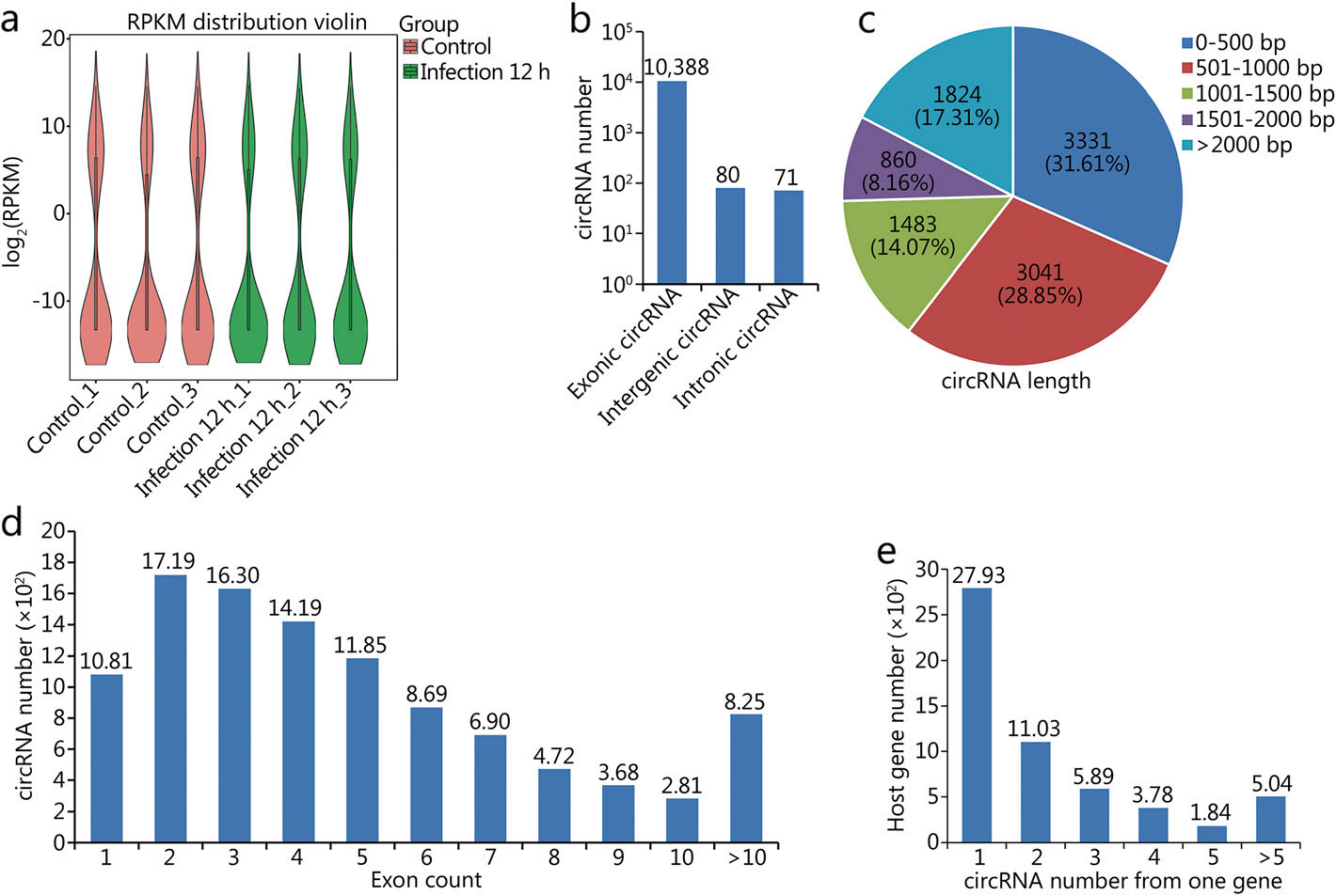
Profiles and characteristics of circRNAs. **a.** Violin plot showing the expression levels of circRNAs in each sample. **b.** The number of circRNAs based on their genomic position. **c.** The length distribution of candidate circRNAs. **d**. The exon counts from one circRNA. **e**. The number of circRNAs transcribed from one gene

Six rRNA-depleted RNA libraries from *T. asahii*-infected and uninfected samples at 12 h were sequenced to analyze the profiles of circRNAs using RNA-seq. The results showed that a total of 10,539 unique circRNAs were identified, among which 4595, 3315 and 4456 circRNAs were identified in the three control samples, and 3422, 4423 and 4091 circRNAs were identified in the three *T. asahii*-infected samples (Fig. 1a). According to the positions of the circRNAs in the genome, we found that exonic circRNAs, totaling 10,388 (98.57%), were the most abundant circRNAs, whereas intronic and intergenic circRNAs totaled only 151 (1.43%) (Fig. 1b). A total of 82.69% (8715/10539) of the circRNAs were less than 2 kp in length, and the average length was approximately 1.54 kb (Fig. 1c). The exon count distribution of the circRNAs showed that most exonic circRNAs (7739, 73.43%) contained more than 2 exons (Fig. 1d). Further, analysis of the circRNAs with respect to their host genes revealed that a great majority of host genes could produce multiple circRNA isoforms (10,539 circRNA candidates from 5551 parental genes) (Fig. 1e).

### Identification of differentially expressed circRNAs

**Fig. 2:**
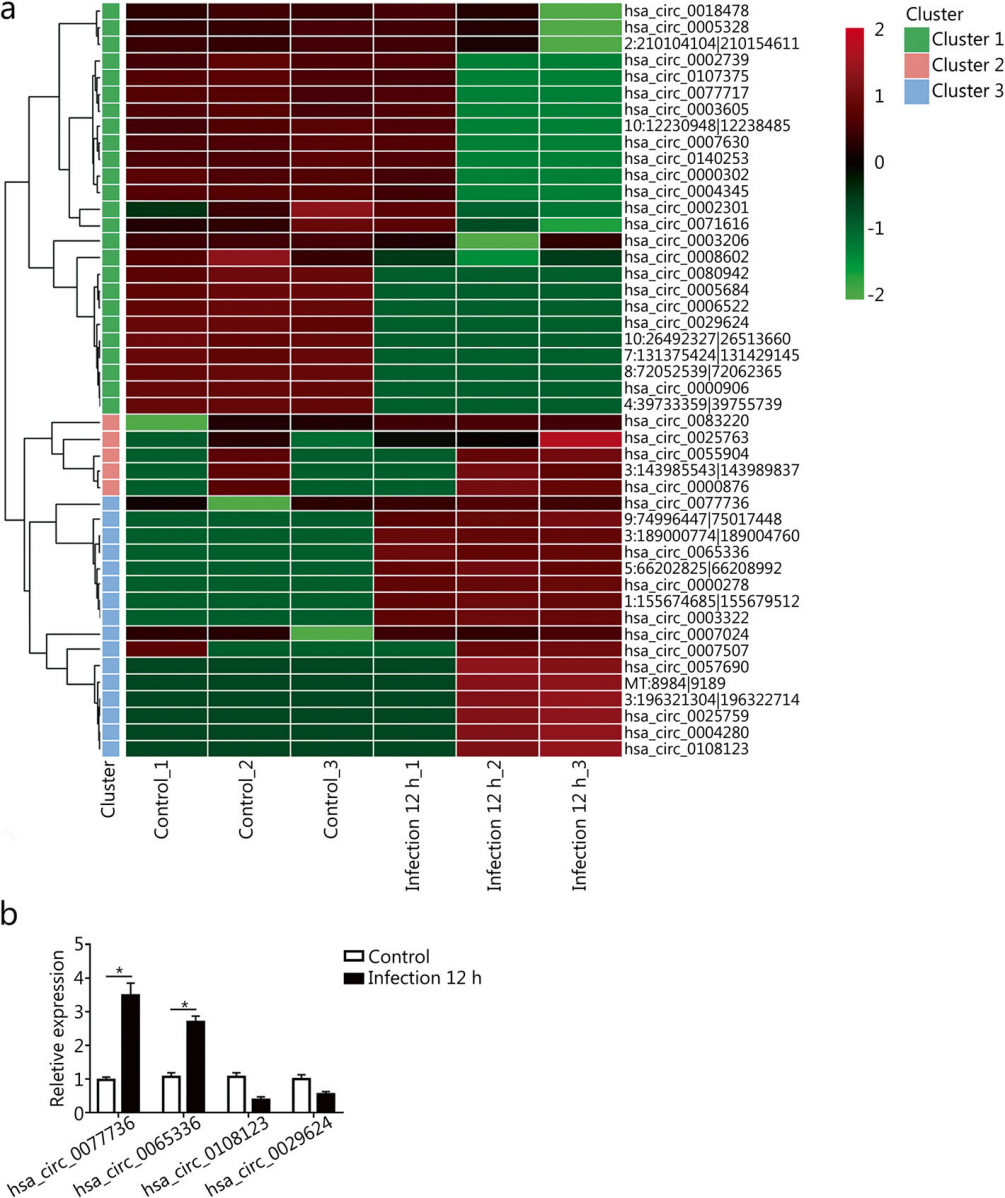
Identification of DE circRNAs after *T. asahii* infection. **a.** Heatmap and clustering analysis of DE circRNAs induced by *T. asahii* in THP-1 cells. **b.** Verification of dysregulated circRNAs by RT-qPCR. ^*^P < 0.05. DE: Differentially expressed

To investigate the differential expression profiles of circRNAs in response to *T. asahii* infection, we employed hierarchical clustering analysis, the results of which indicated that the circRNA expression profiles were significantly changed after *T. asahii* infection. A total of 46 circRNAs were significantly dysregulated in THP-1 cells at 12 h after infection, with 19 up-regulated and 27 down-regulated circRNAs (Fig. 2a and Table S2). To verify the the RNA-seq results, we randomly selected 4 significantly differentially expressed circRNAs (hsa_circ_0077736, hsa_circ_0065336, hsa_circ_0108123 and hsa_circ_0029624) for RT-qPCR verification. The RT-qPCR results showed that their expression followed a trend consistent with the RNA-seq results (Fig. 2b).

### Identification of differentially expressed mRNAs

**Fig. 3:**
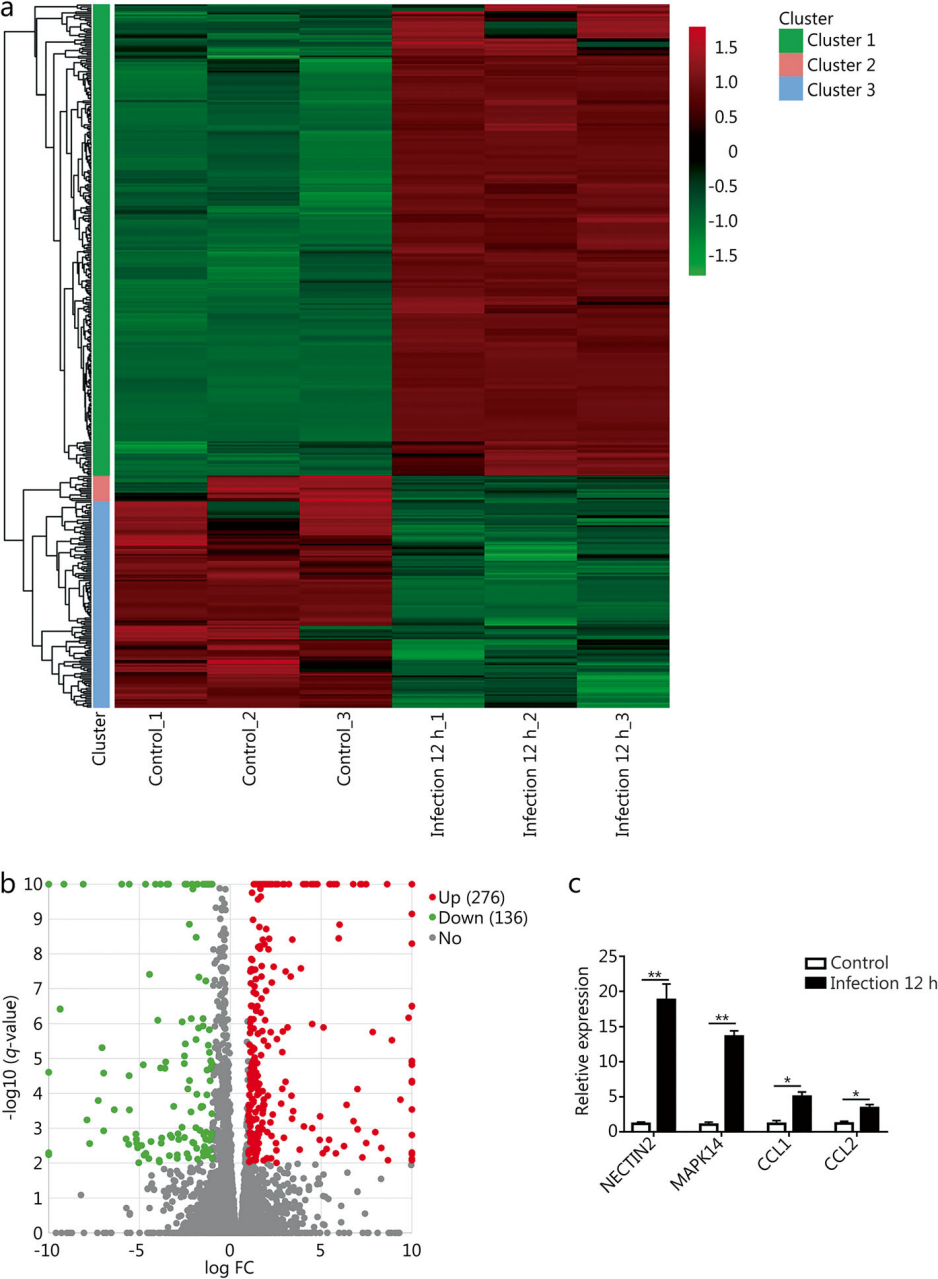
Identification of DE mRNAs in the *T. asahii*-infected and uninfected groups. **a.** Heatmap and clustering analysis of DE mRNAs. **b.** Volcano plot of DE mRNAs at 12 h afte infection against control groups. **c.** Verification of DE mRNAs by RT-qPCR. ^*^P < 0.05; ^**^P < 0.01. FC. Fold change; DE: Differentially expressed

We also examined changes in mRNA expression under the same conditions. A cluster heatmap revealed the differential expression patterns of mRNAs in THP-1 cells after *T. asahii* infection compared with controls. A total of 95,139 mRNAs were identified, and 412 mRNAs were differentially expressed between the two groups; among these mRNAs, 276 were up-regulated, and 136 were down-regulated (Fig. 3a, b and Table S3). To further confirm the RNA-seq results, 4 differentially expressed mRNAs (NECTIN2, MAPK14, CCL1 and CCL2) were randomly selected and validated with RT-qPCR. The results were highly consistent with the RNA-seq data (Fig. 3c).

### Identification of differentially expressed miRNAs

**Fig. 4:**
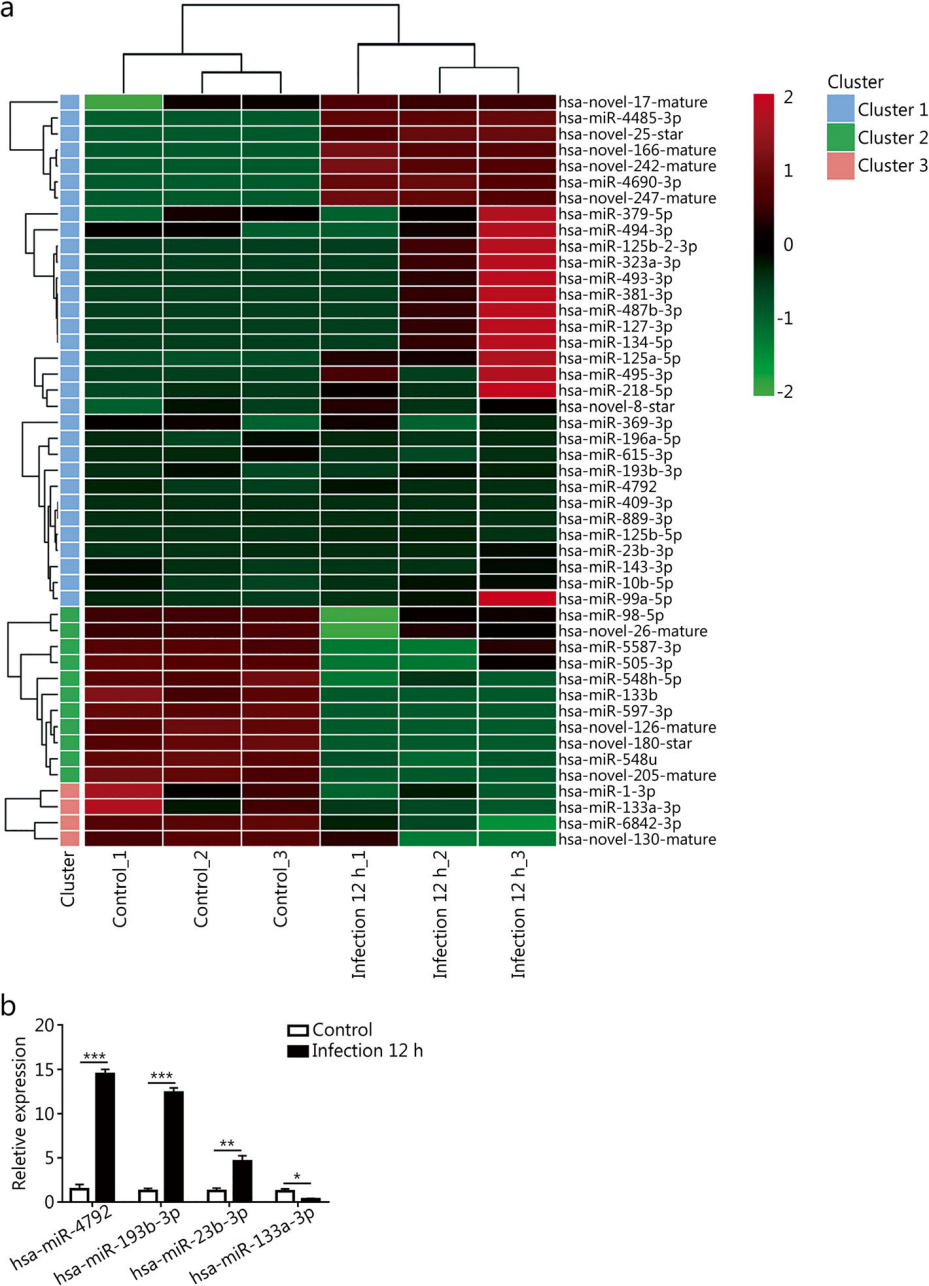
Identification of DE miRNAs upon *T.asahii* infection in THP-1 cells. **a.** Heatmap and clustering analysis of DE miRNAs. **b.** Verification of dysregulated miRNAs by RT-qPCR. ^*^P < 0.05; ^**^P < 0.01; ^***^P < 0.001. DE: Differentially expressed

We further analyzed the differentially expressed miRNAs from the same six samples. A total of 3218 miRNAs were detected, among which 47 miRNAs were significantly dysregulated between the two groups, including 32 up-regulated and 15 down-regulated miRNAs (Fig. 4a and Table S4). Four differentially expressed miRNAs (miR-4792, miR-193b-3p, miR-23b-3p and miR-133a-3p) were randomly selected and verified by RT-qPCR. As expected, the RT-qPCR results were consistent with those of RNA-seq analysis (Fig. 4b).

### GO enrichment and KEGG pathway analyses of differentially expressed mRNAs

**Fig. 5:**
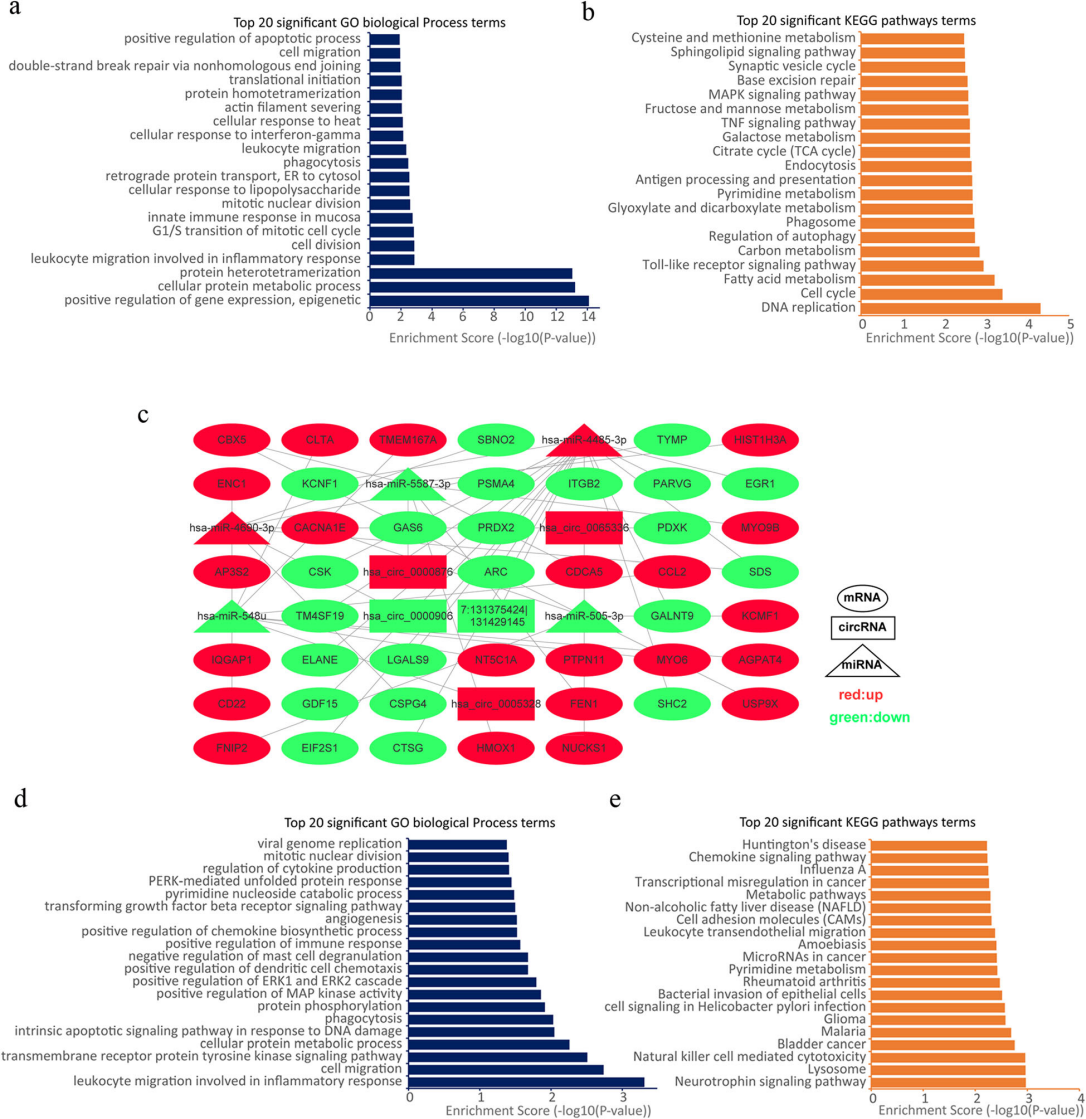
Functional analysis and circRNA-miRNA-mRNA interaction network. **a** and **b** show the top 20 GO biological process terms and KEGG pathway terms of DE mRNAs, respectively. **c.** ceRNA co-expression network. **d** and **e** show the top 20 GO biological process terms and KEGG pathway terms of the target mRNAs of the ceRNA networks. GO: Gene Ontology; KEGG: Kyoto Encyclopedia of Genes and Genomes

We performed GO enrichment and KEGG pathway analyses of the differentially expressed mRNAs. The results of GO enrichment analysis showed that the differentially expressed mRNAs were highly enriched in some biological processes, such as leukocyte migration involved in the inflammatory response, the innate immune response in mucosa, leukocyte migration and phagocytosis (Fig. 5a). In addition, KEGG pathway analysis indicated that multiple mRNAs are primarily involved in the Toll-like receptor (TLR) signaling pathway, phagosome, endocytosis and the TNF signaling pathway (Fig. 5b). These data implied that the cellular antifungal response had been activated by *T. asahii* infection.

### Analysis of the circRNA-miRNA-mRNA regulatory network

Recent studies have shown that circRNAs can indirectly regulate gene expression by acting as a miRNA sponge through a ceRNA mechanism. Therefore, we constructed a circRNA-miRNA-mRNA network according to the ceRNA hypothesis. The screening criteria are listed in the Methods section. Forty-four circRNA-miRNA-mRNA pathways including 5 circRNAs, 5 miRNAs, and 42 mRNAs were established (Fig. 5c and Table S5). Because so many mRNAs are involved in the ceRNA network, we performed GO enrichment and KEGG pathway analyses of the above mRNAs. The results of GO enrichment analysis showed that the differentially expressed mRNAs in the ceRNA network are mainly involved in leukocyte migration involved in the inflammatory response, the transmembrane receptor protein tyrosine kinase signaling pathway and phagocytosis (Fig. 5d). Furthermore, KEGG pathway analysis indicated that the neurotrophin signaling pathway, lysosome, and natural killer cell mediated cytotoxicity are the main pathways in which these mRNAs are involved (Fig. 5e).

### Verification of the ceRNA network

**Fig. 6:**
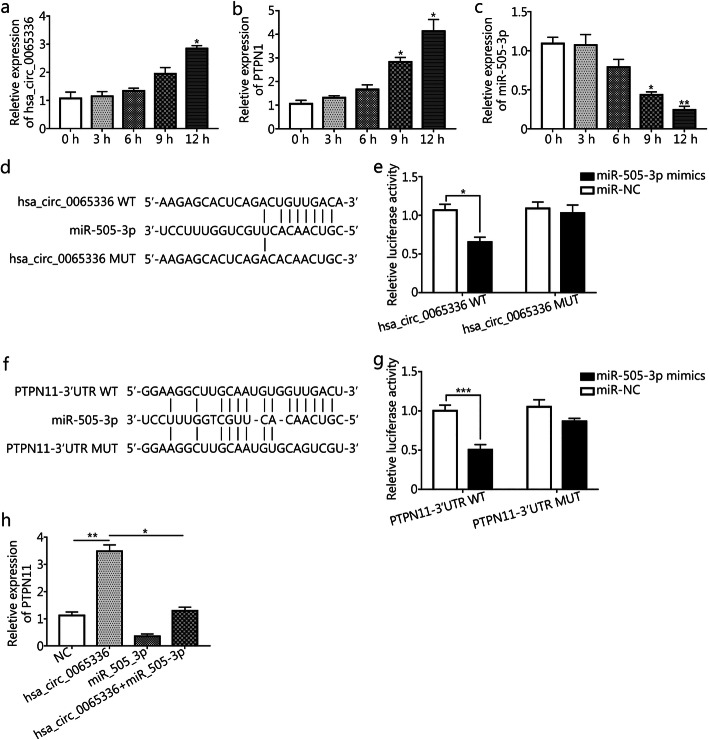
Verification of the hsa_circ_0065336/miR-505-3p/PTPN11 regulatory pathway. **a-c.** The mRNA expression levels of hsa_circ_0065336 (**a)**, miR-505-3p (**b)** and PTPN11 (**c)** were determined by RT-qPCR at the indicated time points. **d.** Putative binding sites of hsa_circ_0065336 and miR-505-3p. **e.** Dual-luciferase reporter assay between hsa_circ_0065336 and miR-505-3p. **f.** Putative binding sites of miR-505-3p and PTPN11. **g.** Dual-luciferase reporter assay between miR-505-3p and PTPN11. **h.** The PTPN11 expression level was regulated by hsa_circ_0065336 and miR-505-3p. ^*^P < 0.05; ^**^P < 0.01. MT: Wild type; MUT: Mutant type; NC: Negative controls

To demonstrate the authenticity of the ceRNA network, we selected the predicted hsa_circ_0065336/miR-505-3p/PTPN11 pathway based on the literature and GO enrichment and KEGG pathway analyses carried out in the previous step. We first measured the expression levels of hsa_circ_0065336, miR-505-3p and PTPN11 in THP-1 cells after infection with *T. asahii* at time points of 0, 3, 6, 9, and 12 h. hsa_circ_0065336 and PTPN11 expression was up-regulated in a time-dependent manner, reaching a peak at 12 h (*P* = 0.033 and 0.029, respectively) (Fig. 6a, b), whereas miR-505-3p expression was down-regulated starting at 3 h after infection (Fig. 6c) and then was maintained at a decreased level at 9 h after infection (*P* = 0.021), which was highly similar to the RNA-seq results.

Bioinformatics prediction analysis based on the miRanda database showed that miR-505-3p targets both hsa_circ_0065336 and PTPN11 (Fig. 6d, f). To confirm this prediction, full-length hsa_circ_0065336-WT and hsa_circ_0065336-MUT were constructed and subcloned into the dual-luciferase report vector pmir-GLO, after which the plasmids were cotransfected with miR-505-3p mimics or NC into 293 T cells. The results showed that hsa_circ_0065336-WT with the miR-505-3p mimics significantly decreased the luciferase activity (*P* = 0.041), whereas hsa_circ_0065336-MUT did not affect the luciferase activity (Fig. 6e). Additionally, the luciferase activity of cells transfected with PTPN11 3’UTR-WT was significantly decreased by transfection with miR-505-3p mimics (*P* = 0.029) (Fig. 6g).

To further verify the hsa_circ_0065336/miR-505-3p/PTPN11 pathway in THP-1 cells after *T. asahii* infection, we transfected an hsa_circ_0065336-overexpression vector into THP-1 cells and subsequently infected these cells with *T. asahii* for 12 h. Compared with the control treatment, overexpression of hsa_circ_0065336 significantly elevated PTPN11 levels (*P* = 0.005). Additionally, the increase in PTPN11 induced by hsa_circ_0065336 overexpression could be reversed by transfection with miR-505-3p mimics (*P* = 0.026) (Fig. 6h). Taken together, these data identified the hsa_circ_0065336/miR-505-3p/PTPN11 pathway in THP-1 cells upon *T. asahii* infection.

## Discussion

Several recent studies have reported circRNA expression profiles and ceRNA networks in viral and bacterial infectious diseases [25–28]. Nevertheless, the expression profiles and potential roles of circRNAs in fungal infection have not been explored.

In this study, we systematically investigated the circRNA, miRNA, and mRNA expression profiles of *T. asahii*-infected THP-1 cells and compared them with those of uninfected samples. circRNA expression, the type and length of circRNAs and host gene number were similar to those in previous reports [29, 30]. We found a total of 46 differentially expressed circRNAs, 412 differentially expressed mRNAs and 47 differentially expressed miRNAs following *T. asahii* infection for 12 h. Previous studies found that several pattern recognition receptors (PRRs), including TLR-2, Dectin-1, galectin-9 and PTX3, are involved in the recognition of diverse pathogen-associated molecular patterns (PAMPs) in *Trichosporon* spp. PRP-PAMP binding could activate a downstream immune signaling pathway, increasing the production of ROS and inflammatory factors, such as TNF-α, resulting in fungal killing [31]. In this study, we also identified a group of genes that participate in the *T. asahii*-host interaction, and GO enrichment and KEGG pathway analyses showed that many of the up-regulated genes, including TNF alpha induced protein 3 (TNFAIP3), TNF receptor superfamily member 10d (TNFRSF10D), TNF receptor superfamily member 1A (TNFRSF1A), CD14, interferon alpha and beta receptor subunit 1 (IFNAR1), interferon regulatory factor 5 (IRF5), were enriched in immune-related pathways, suggesting that *T. asahii* infection can activate a strong antifungal response that contributes to fungal pathogenic progression. These findings are highly consistent with those of previous reports and reveal a more comprehensive gene regulatory network.

The ceRNA hypothesis first proposed by Salmena et al. [32] in 2011 describes how mRNAs, transcribed pseudogenes, and long noncoding RNAs (lncRNAs) “talk” to each other using MREs as letters in a new language. Many studies have found that circRNAs can act as ceRNAs to sponge miRNAs, indirectly regulating the expression of genes and participating in the occurrence and development of various diseases, especially cancer. Since no data about circRNA-associated ceRNAs in fungal infections have been reported, we systematically identified a circRNA-miRNA-mRNA regulatory network after *T. asahii* infection based on the RNA-seq and ceRNA criteria. Five circRNAs, 5 miRNAs, and 42 mRNAs were screened and used to construct a ceRNA regulatory network. Furthermore, we performed GO and KEGG analyses of the 42 predicted target mRNAs in the ceRNA network to explore the functions of the five circRNAs. The results showed that many host immune response pathways, including the inflammatory response, phagocytosis and the neurotrophin signaling pathway, were involved, indicating that circRNAs may be the principal regulators of the host response during *T. asahii* infection.

Moreover, we selected the hsa_circ_0065336/miR-505-3p/PTPN11 pathway to verify the reliability of the ceRNA network using RT-qPCR, dual-luciferase reporter assays and overexpression experiments. PTPN11 is a cytoplasmic tyrosine phosphatase that participates in growth factor, cytokine and hormone signaling. A recent study found that PTPN11 is crucial for the induction of pro-inflammatory cytokines and the control of *Candida albicans* infection through the Dectin-1/PTPN11/Syk/NF-κB pathway [33]. miR-505-3p has been shown to be down-regulated in several autoimmune diseases, including primary biliary cirrhosis and inflammatory bowel disease [34, 35]. Escate et al. [36] reported that miR-505-3p is decreased in familial hypercholesterolemia, which up-regulated several chemokine receptors by targeting the RUNX1 transcription factor in macrophages. hsa_circ_0065336 expression has been reported in the brain and several cell types, but no study has assessed its function in any disease or physiological process. Here, we found that hsa_circ_0065336 and PTPN11 were simultaneously up-regulated, whereas miR-505-3p expression was down-regulated, in *T. asahii-*infected THP-1 cells. Dual-luciferase reporter assays demonstrated that miR-505-3p could directly target hsa_circ_0065336 and PTPN11. Moreover, a functional assay revealed that PTPN11 expression was up-regulated by hsa_circ_0065336 but inhibited by miR-505-3p. Therefore, the hsa_circ_0065336/miR-505-3p/PTPN11 regulatory axis upon *T. asahii* infection was identified and verified for the first time.

## Conclusions

As far as we know, this is the first report to jointly analyze the expression profiles of circRNAs, miRNAs and mRNAs upon fungal infection in mammalian cells. We constructed a circRNA-associated ceRNA regulatory network, the components of which are mainly involved in the fungal-host immune response. In conclusion, this study can provide novel insight into the pathogenesis of *T. asahii* infection.

## Supplementary Information

**Additional file 1: Table S1.** Primers used to assess select circRNAs, mRNAs and miRNAs by RT-qPCR. **Table S2.** Differentially expressed circRNAs. **Table S3.** Differentially expressed mRNAs. **Table S4.** Differentially expressed miRNAs. **Table S5.** The circRNA-miRNA-mRNA regulatory network.

## Data Availability

The datasets used and/or analysed during the current study are available from the corresponding author on reasonable request.

## Abbreviations

ceRNA: Competing endogenous RNA
circRNA: Circular RNA
GO: Gene Ontology
IFNAR1: Interferon alpha and beta receptor subunit 1
IRF5: Interferon regulatory factor 5
KEGG: Kyoto Encyclopedia of Genes and Genomes
miRNA: MicroRNA
lncRNA: Long noncoding RNA
MOI: Multiplicity of infection
MREs: miRNA response elements
MT: Wild type
MUT: Mutant type
NC: Negative controls
PAMPs: Pathogen-associated molecular patterns
PBS: Phosphate-buffered saline
PMA: Phorbol 12-myristate 13-acetate
PRPs: Pattern recognition receptors
RNA-seq: RNA sequencing
RPKM: Reads per kilobase of transcript per million mapped reads
RPM: Reads per million
RT-qPCR: Real-time quantitative PCR
*T. asahii*: *Trichosporon asahii*
TLR: Toll-like receptor
TNFAIP3: TNF alpha induced protein 3
TNFRSF10D: TNF receptor superfamily member 10d
TNFRSF1A: TNF receptor superfamily member 1A
TPM: Transcripts per million
YPD: Yeast extract peptone dextrose
